# Rapid Copper Acquisition by Developing Murine Mesothelioma: Decreasing Bioavailable Copper Slows Tumor Growth, Normalizes Vessels and Promotes T Cell Infiltration

**DOI:** 10.1371/journal.pone.0073684

**Published:** 2013-08-27

**Authors:** Andrew Crowe, Connie Jackaman, Katie M. Beddoes, Belinda Ricciardo, Delia J. Nelson

**Affiliations:** 1 Immunology and Cancer Group, School of Biomedical Sciences, Curtin University, Bentley, Perth, Western Australia, Australia; 2 School of Pharmacy, CHIRI Biosciences Research Precinct, Curtin University, Bentley, Perth, Western Australia, Australia; 3 School of Biomedical Sciences, CHIRI Biosciences Research Precinct, Curtin University, Bentley, Perth, Western Australia, Australia; University of Kentucky, United States of America

## Abstract

Copper, an essential trace element acquired through nutrition, is an important co-factor for pro-angiogenic factors including vascular endothelial growth factor (VEGF). Decreasing bioavailable copper has been used as an anti-angiogenic and anti-cancer strategy with promising results. However, the role of copper and its potential as a therapy in mesothelioma is not yet well understood. Therefore, we monitored copper levels in progressing murine mesothelioma tumors and analyzed the effects of lowering bioavailable copper. Copper levels in tumors and organs were assayed using atomic absorption spectrophotometry. Mesothelioma tumors rapidly sequestered copper at early stages of development, the copper was then dispersed throughout growing tumor tissues. These data imply that copper uptake may play an important role in early tumor development. Lowering bioavailable copper using the copper chelators, penicillamine, trientine or tetrathiomolybdate, slowed in vivo mesothelioma growth but did not provide any cures similar to using cisplatin chemotherapy or anti-VEGF receptor antibody therapy. The impact of copper lowering on tumor blood vessels and tumor infiltrating T cells was measured using flow cytometry and confocal microscopy. Copper lowering was associated with reduced tumor vessel diameter, reduced endothelial cell proliferation (reduced Ki67 expression) and lower surface ICAM/CD54 expression implying reduced endothelial cell activation, in a process similar to endothelial normalization. Copper lowering was also associated with a CD4^+^ T cell infiltrate. In conclusion, these data suggest copper lowering is a potentially useful anti-mesothelioma treatment strategy that slows tumor growth to provide a window of opportunity for inclusion of other treatment modalities to improve patient outcomes.

## Introduction

The realization that angiogenesis is essential for tumor growth, invasion and metastasis led to the development of anti-angiogenic therapies [1]. A number have been tested, however, toxicity issues thwarted potentially promising outcomes [2]. Strategies that target vascular endothelial growth factor (VEGF) can be transiently successful until sabotaged by tumor up-regulation of other pro-angiogenic factors [3]. Copper (Cu), a trace metal involved in many essential processes, such as energy metabolism and hemoglobin production, also plays an integral role in tumor angiogenesis by functioning as a critical co-factor for several pro-angiogenic molecules including VEGF, basic fibroblast growth factor (bFGF), and angiogenin [4,5]. Therefore, therapeutic copper reduction achieved by lowering the levels of bioavailable copper using copper chelators, represents an anti-cancer approach that targets multiple pro-angiogenic factors and is reported to be relatively non-toxic [6,12].

Importantly, decreasing bioavailable copper has shown promising results in animal models and in clinical trials in different cancers [6–10], including a single post surgical trial in patients with malignant mesothelioma [11]. Copper lowering may modulate tumor blood vessels to normalize them, i.e. render them more phenotypically, structurally and functionally similar to vessels in normal healthy tissue, as demonstrated in a brain cancer rabbit model [12]. In that model, lowering the concentration of local copper reduced the abnormally high proliferative rate of tumor endothelia to the more quiescent rate seen in normal brain tissue [12]. Furthermore, tumor vessel width was reduced to a similar width seen in the healthy brain vessels. Beneficial consequences of vessel normalization may be facilitation of immune cell infiltration and/or enhanced immune cell function [13–15]. We address these issues using a murine mesothelioma model.

Malignant mesothelioma is characterized by an abnormal proliferation of serosal-surface mesothelial cells and is associated with asbestos exposure [16]. Mesothelioma patients have a mean survival of 12 months after diagnosis and the incidence is rising worldwide due to past, recent and in some cases current exposure to asbestos. The poor prognosis of this cancer on account of resistance to conventional treatment strategies including surgery, chemotherapy and radiation, warrants the search for alternative treatment options. The present study determines (i) if and when developing mesothelioma tumors take up copper, and (ii) if copper-lowering strategies affect tumor vasculature, tumor growth and immune cell infiltration.

## Materials and Methods

### Mice

Female C57BL/6J mice aged 6 to 8 weeks obtained from the Animal Resource Centre (Perth, Western Australia (WA) were maintained under Specific Pathogen Free conditions in the Curtin University animal holding facility. All mouse experiments were performed according to the Australian Code of Practice for the care and use of animals for scientific purposes as per Curtin University Animal Ethics Committee (AEC) approval numbers AEC-2011-01 and AEC-2011-16. Mice were provided a standard meat free rat and mouse diet (Specialty Feed, Perth, Western Australia) ad libitum which, according to the manufacturer, contains 0.0016% copper or 16 µg copper per gram chow.

### Cell lines and in vivo tumor growth

AE17 is a mesothelioma cell line derived in C57BL/6J mice after intraperitoneal (i.p.) inoculation of asbestos fibres [17]. AE17 cells were maintained at 37 °C in 5% CO_2_ in RPMI 1640 media (Invitrogen, CA, USA) supplemented with 10% fetal calf serum (FCS) (Invitrogen, CA, USA), 50 µg/ml gentamicin (Sigma Aldrich, USA) and 100 U/ml penicillin (Sigma Aldrich). At 80% confluency, cells were trypsinized, washed with PBS (Invitrogen) and cell numbers determined using trypan blue (Sigma Aldrich). Subcutaneous (s.c.) inoculation of 5 × 10^5^ AE17 cells in 100 µl of PBS into mice produces tumors histologically similar to human mesothelioma [17]. Tumor sizes were determined by multiplying two perpendicular axes that are at right angles to each other (measured using microcallipers) to get an area calculation. Note that tumor volumes were not determined as the tumors do not reliably develop into spherical, ellipsoid, or hemi-ellipsoid shapes that have defined formulae to use. Human umbilical vein endothelial cells (HUVECs, ATCC-CRL-1730; American Type Culture Collection, VA, US) were maintained in endothelial cell medium (ECM) supplemented with endothelial cell growth supplement, foetal bovine serum penicillin and streptomycin (all from ScienCell Research Laboratories) at 37°C in 5% CO_2_.

### Using the MTT assay to measure cell viability and proliferation

The 3-(4,5-Dimethyl-2-thiazolyl)-2,5-diphenyl-2H-tetrazolium bromide (MTT) assay is based on the cleavage of the yellow tetrazolium salt (MTT) to purple formazan crystal by metabolically active cells and is used to measure the level of energy metabolism occurring in cells, as well as cell viability and cell proliferation. Previously identified optimal concentrations (5 x 10^3^) cells/well were seeded in flat bottom 96-well plates (Becton Dickinson (BD) USA) and incubated in 200 µl/well for 24 hrs when 50 µl of 2mg/ml MTT (Sigma Aldrich) in PBS was added. Cells were incubated at 37°C for 4 hrs, centrifuged at 1,000 rpm at 4°C for 5 mins, supernatant removed and 100 µl of dimethyl sulfoxide (*DMSO*; Sigma Aldrich) added to each well. Plates were incubated in the dark for 30 mins at room temperature (RT) with shaking and absorbance determined using a Biorad 3550 microplate reader at 595 nm.

### Cell cycle analysis

Staining DNA in intact ethanol-fixed cells with propidium iodide (PI) can be used to determine which phase of the cell cycle (G0, G1, S, G2 or M phase) a cell is in after exposure to a specific treatment. PI, a fluorescent dye, passes through the ethanol-permeabilized cell membrane and is incorporated into the DNA. The intensity of the PI signal is directly proportional to DNA content which identifies the cell cycle phase. AE17 cells were seeded at 5 x 10^5^ cells/well using 6-well plates (BD), incubated for 24 hrs, given the relevant treatment, and incubated for a further 24 hrs. Cells were trypsinized, harvested, centrifuged at 1,350 rpm for 10 mins then re-suspended in glucose buffer (1g glucose/L PBS) and washed twice in glucose buffer prior to overnight (O/N) fixing in 70% ethanol at 4°C. Fixed cells re-suspended in 1 ml of a 50 mg/ml (PI; Sigma Aldrich) *solution* in glucose buffer containing 2 mg/ml ribonuclease A (Sigma Aldrich) were incubated in the dark at RT for 30 mins prior to flow cytometric analysis.

### In vivo copper depletion and targeting VEGF in vivo

Mice offered copper-free milli-Q water (Ibis Ultra Pure water system) and mouse chow (Able Scientific, WA) ad libitum were given penicillamine, trientine or tetrathiomolybdate(TM) (Sigma Aldrich). Penicillamine and trientine deplete copper by binding free copper in blood and tissues, creating a stable soluble complex that is excreted in urine [18]. Penicillamine is a reductive copper chelator that lowers the affinity of copper for protein and allows it to be chelated [19]. Trientine increases the excretion of copper and decreases intestinal copper absorption [20]. TM forms a stable tripartite complex with copper and serum albumin, rendering it unavailable for cellular uptake, this complex is slowly cleared in bile and urine [4]. Low, intermediate and high doses based on previous studies [5,6,18,21,22] were administered via i.p. injection in 200 µl of copper-free Milli-Q water. Mice were monitored for behavioral or physical changes that indicate toxicity. In one series of experiments, VEGF was targeted using the anti-VEGF-receptor(r) monoclonal antibody, DC101 [23], purchased from the Western Australian Medical Institute for Medical Research (WAIMR) Monoclonal Antibody Facility (Perth, Western Australia). Mice were given 3 i.p. doses (800 µg in 800 µl) of the anti-VEGFr antibody every 3 days as previously described [23].

### Harvesting tissues for copper analysis

After collecting blood via cardiac puncture using a heparinized (David Bull Laboratories, UK) 1 ml syringe (BD, CA, USA) mice were perfused with 15 mls of cold PBS containing 10 units/ml of heparin until the organs blanched. Organs and tumors were collected, washed twice in Milli-Q water, wet weights recorded and frozen. Upon thawing, samples were homogenized using a Heidolph DIAX 900 into 8 volumes of water (e.g. 100 µg in 800 µl). A 300 µl aliquot was lyophilised (Edwards EF4 Modulyo Freeze dryer) for 5 hrs, dry weights recorded and then digested in a 2:1.6 (vol: vol) mixture of 70% Nitric Acid (Sigma Aldrich) and 30% hydrogen peroxide (Sigma Aldrich) and heated to 100°C for one hour as described [24]. Digested samples were diluted to 4 mls with Milli-Q water and blood samples were diluted 1:200 in 1% Triton–X-100.

### Determining copper levels

Atomic absorption spectrophotometric (AAS) analysis was used to determine the concentration of copper in digested tissue and blood samples. A hollow copper cathode lamp operating at 4 mA was used, and the spectrophotometer set to read absorbance at 327.4 nm with a spectral bandwidth of 0.5nm. Standard curves ranging from 0 µg/L to 120 µg/L were created using a copper nitrate, Cu(NO_3_)_2_ standard (Varian, Australia). Using the spectraAA 640Z (Varian, Australia) with a GTA-100 autosampler, 20 µl of each processed sample was injected into a graphite tube (Varian, Australia), which was heated according to the protocol outlined in Table 1. Each sample was measured in triplicate, the mean absorbance was compared to the standard curve and the copper concentration calculated after background correction was performed using an inbuilt Zeeman apparatus. A second machine was also used, GBC Avanta 908G, with the same parameters as described above.

**Table 1:** Furnace Parameters used in Atomic Absorption Spectrophotometry.

Temp (°C)	Time (seconds)*	Gas Flow (L/min)	Gas type	Read absorbance
80	6	3.0	Nitrogen	No
90	40	3.0	Nitrogen	No
110	11	3.0	Nitrogen	No
800	5	3.0	Nitrogen	No
800	1	3.0	Argon	No
800	2	0	Argon	No
2300	1.1	0	Argon	Yes
2300	2	0	Argon	Yes
2300	2	3.0	Nitrogen	No

* Values refer to ramping time, the time taken to increase temperature within the graphite tube from previous temperature to the current temperature.

AE17 cells were digested in 300 µl of 70% HNO_3_ (Riedel-de Haën, Germany) at 90°C for 30 mins and 200 µl of 40% H_2_O_2_ (Sigma Aldrich) for 10 mins at 70°C until the solution became clear. Milli-Q water was added to give a final volume of 5 mls and copper levels measured using inductively coupled plasma-mass spectrometry (ICP-MS; TSW Analytical Pty Ltd, Biomedical, University of WA, Perth, Australia).

### Determining plasma ceruloplasmin levels

The majority of serum copper is transported bound to ceruloplasmin thus it can be used as a surrogate maker for blood copper levels [25–27]. A 25 µl sample of plasma was added to 375 µl of 0.1 mol/L sodium acetate (pH 5), incubated at 30 °C for 5 mins and 100 µl of substrate (O-dianisidine dihydrochloride) (Sigma Aldrich) added. One of a duplicate sample was incubated at 30 °C for 25 mins (described as A_25_ in the formula below) whilst the remaining duplicate was incubated for 40 mins (A_40_). This assay determined the enzymatic activity of the ceruloplasmin between the two time points by its oxidation of the substrate to a yellow product. One ml of 9 mol/L sulfuric acid (H_2_SO_4_) was added to quench the reaction and form a stable pink product, which was analyzed at 540nm. Ceruloplasmin activity was determined using the following equation:

Ceruloplasmin Activity IU/ml = (A_40_-A_25_) × 6.25×10^-1^ (Where 0.625 is a factor that takes into account dilution factor of 60, incubation of 10, light path of 1 cm, and an absorptivity of 9.6 µmol/ml).

### Harvesting tumors and lymphoid organs

Spleens, draining (dLN) and non-draining lymph nodes (ndLN) were disaggregated into single cell suspensions by gently mashing between two frosted slides, removing debris, centrifugation at 1200 rpm for 5 mins and re-suspension in 2% FCS in PBS.

### Assessing immune cells and blood vessels using flow cytometric analysis

Cell samples were incubated in the dark for 30 mins at 4°C with 50 µl buffer containing the antibodies (Ab) described below, washed twice in PBS/2% FCS and re-suspended in 2% paraformaldehyde for flow cytometry analysis. Markers for mouse endothelial cells were anti-CD31 (PECAM; Biolegend, San Diego, CA, USA); Alexa Fluor^®^ 647-conjugated-anti-CD34 (found on normal endothelial cells and stem cells [28]), PE-anti-CD105 (Endoglin, a regulatory component of TGF-β receptor specific for tumor angiogenesis [28]), Alexa Fluor^®^ 647-anti-CD106 (vascular cell adhesion molecule; VCAM) expressed on activated endothelial cells; all from eBioscience, San Diego, CA, USA. FITC-anti-CD54 (intracellular adhesion molecule-1, ICAM-1; BD Pharmingen, CA, USA) is expressed on activated endothelial cells. Ki67 is an intracellular marker of proliferation. Surface staining was first performed as described above. Cell membranes were then fixed and permeabilised using fixation/permealisation buffer (Biolegend), washed and further incubated with permealisation buffer (Biolegend) as per manufacturer’s instructions. The cells were then incubated with FITC-anti-Ki67 (BD Pharmingen) for 30 mins in the dark. For immune cell analysis single cell suspensions were co-stained with APC Cy7-anti-CD3 and PE-anti-CD8 (both from BD Pharmingen), as well as PECy5.5-anti-CD4 and/or PE-anti-CD19 (both from Biolegend), and FACS analyzed. All flow cytometry data was acquired on a FACS Canto II using FACS Diva Software. FlowJo software was used to analyze the data.

### Assessing blood vessels using immunofluorescence

Frozen sections (10 µm) of OCT-embedded tumors were fixed in cold acetone for 10 mins, air dried, and treated with PBS/5% FCS for 30 mins at RT. FITC-anti-CD31 (PECAM; Biolegend) or isotype control (rat IgG2a; BD, Mountain View, CA) were applied for 45 mins at RT. Sections were rinsed in PBS/5% FCS and mounted using VectaShield (Vector Laboratories, Burlingame, CA, USA). Slides were visualized on an UltraVIEW VoX confocal imaging system with Volocity 6.0.1 software (PerkinElmer, Massachusetts, USA). Blood vessel measurements were calculated using Volocity 6.0.1 software.

### Statistical analysis

The student t-test and one-way analysis of variance (ANOVA) were used to determine differences between two or more populations respectively using GraphPad PRISM 4.0. Differences were considered significant if p < 0.05.

## Results

### Tap water is a source of consumed copper

Copper levels in drinking water were measured to address the significance of water contributing to overall dietary copper absorption. Tap water contained high copper levels (Figure S1A) that were significantly reduced by filtration and ultra purification (Milli-Q) methods. Mice maintained on tap water appeared to contain higher levels of copper in livers and kidneys than mice maintained on Milli-Q water (Figure S1B) however, the differences did not reach statistical significance. These data suggest that food is a significant source of copper. Nonetheless, mice in all of the following experiments were maintained on Milli-Q filtered water and the copper chelating reagents were diluted in Milli-Q water to reduce copper consumption.

### Mesothelioma tumors rapidly sequester copper in vivo

**Figure 1:**
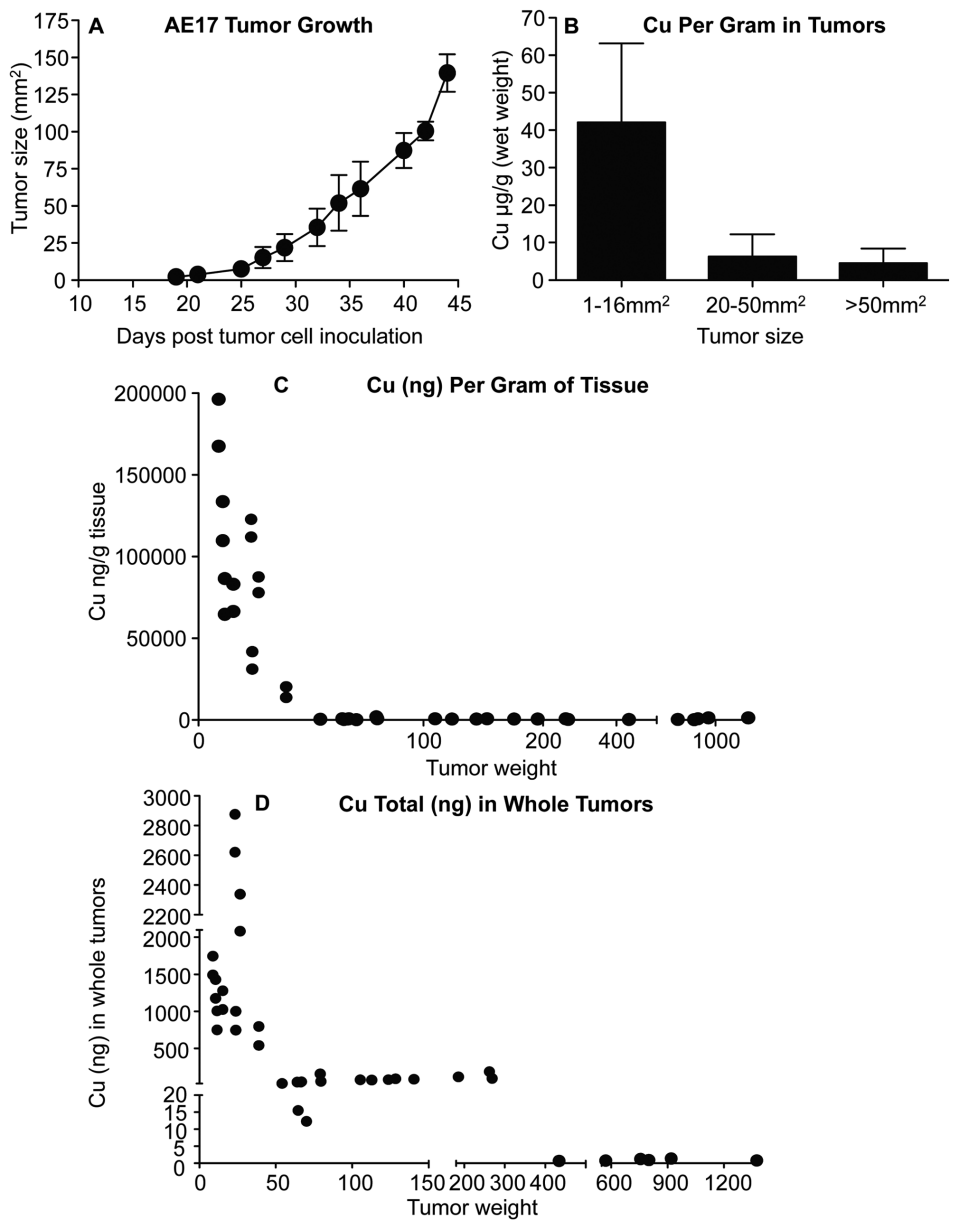
Developing mesothelioma tumors rapidly acquire copper. Mice were sacrificed when tumors reached 1-16 mm^2^, 20-50 mm^2^ and > 50 mm^2^. Tumor growth rate shown in A. Copper levels from the different sized tumors (B); pooled data from 4 experiments, with n = 19 mice with 1-16mm^2^ tumors; n = 10 mice with 20-50mm^2^ tumors; n = 16 mice with > 50mm^2^ tumors; and n = 10 control mice is shown as mean ± SEM. Copper levels are also shown as individual weighed tumor samples to better reflect tumor size (C). Cu levels in whole tumor tissue relative to individual tumor weights are also shown (D).

Copper levels in tumors and tissues were measured during mesothelioma tumor growth (Figure 1A). Copper analysis on a per gram basis in terms of tumor size (Figure 1B), or tumor weight (Figure 1C), shows that mesothelioma tumors had their greatest concentrations at early stages of tumor evolution with concentrations over 5 times that found in livers and kidneys (Figure S2). Total copper levels (Figure 1D) and copper levels per gram (Figure 1B and C) then dropped considerably, suggesting that accumulated copper was dispersing throughout growing tumor tissues faster than their ability to mobilize copper into the tumors.

### Mesothelioma tumors do not induce changes to copper levels in other organs

No significant changes in copper concentrations in livers, kidneys, lungs and spleens (Figure S2A, S2B, S2C and S2D respectively) were seen in mesothelioma-bearing mice during disease progression. The levels in these organs are similar to those previously reported by others [24,26], thus these tissues also functioned as a useful internal control.

### Bioavailable copper levels are reduced in vivo using copper lowering agents

**Figure 2:**
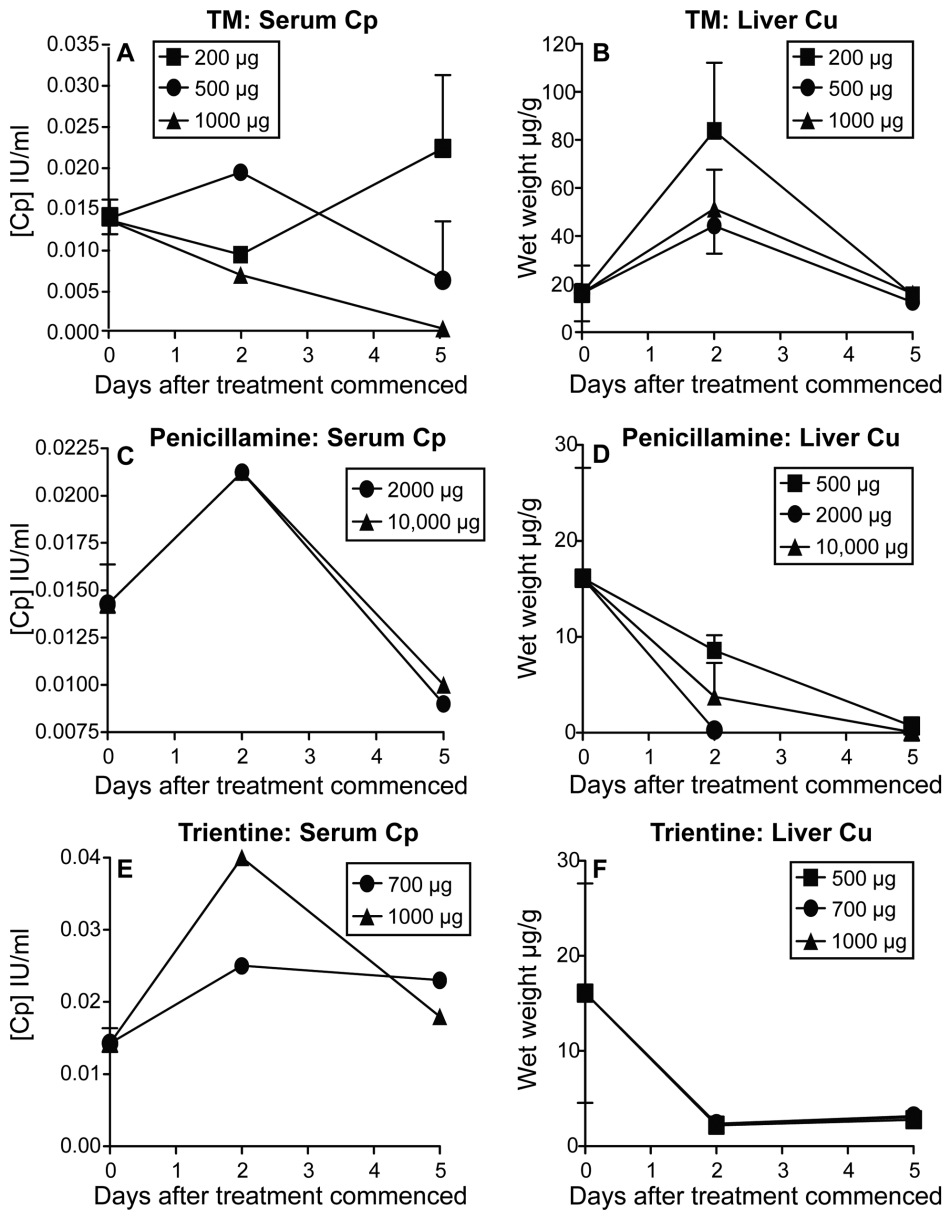
Bioavailable copper levels can be decreased in vivo by copper chelating agents. Low, medium and high doses of TM (A and B), penicillamine (C and D) and trientine (E and F) were administered i.p. to healthy mice over 5 days. Mice were sacrificed on days 0, 2 and 5 Plasma ceruloplasmin (Cp) was assayed (A, C and E), and liver Cu levels analyzed (B, D and F). For Cu levels n = 3 mice/group. For Cp assays there were 11 mice for TM at day 0, these mice were divided into groups and given different doses of TM; 1 mouse/TM dose was culled and sampled at day 2 (n = 1/group) for each of the three doses; 3 mice were sampled for each of the 200 µg and 1000µg TM doses (n = 3/group), and 2 mice sampled for the 500µg TM treatment at day 5 (n = 2/group). For penicillamine and trientine there were 4 mice per treatment dose at day 0 (n = 4) and 1 mouse sampled at each time point for each dose (n = 1/group). Where possible, data is shown as mean ± SEM.

We then aimed to identify an in vivo treatment protocol that would reduce bioavailable copper levels. Penicillamine [29–31], trientine [32–34] and TM [9,35,36] have been described as effective copper lowering agents, hence we analyzed their effectiveness in 6 week old mice weighing between 21 and 23 grams. The high TM dose (1000 µg per mouse) and both penicillamine doses (2000 µg and 10,000 µg per mouse) led to a decrease in plasma ceruloplasmin levels 5 days after treatment relative to Day 0 ceruloplasmin levels taken before treatment commenced (Figure 2A and C). Both doses of trientine (700 µg and 1000 µg per mouse) appeared to transiently increase ceruloplasmin levels at day 2 yet they returned to baseline levels by day 5 (Figure 2E). All doses of trientine and penicillamine treatment caused a decrease in liver copper levels (Figure 2D and F). As ceruloplasmin is used as surrogate marker for blood copper levels the results suggest that both penicillamine and trientine mobilize copper into blood before eliminating it, and liver copper levels decreased rapidly with trientine treatment and more slowly with penicillamine treatment. In contrast, liver copper levels in TM treated-mice transiently increased before decreasing to below day 0 levels (Figure 2B) suggesting the mobilization and accumulation of copper in the liver before its excretion. Whilst no macroscopic signs of toxicity were observed at any dose used, the doses chosen for subsequent experiments were: TM 500 µg per dose per mouse; trientine 700 µg per dose per mouse; penicillamine 2000 µg per dose per mouse.

### Therapeutically lowering bioavailable copper slows mesothelioma tumor growth rate

**Figure 3:**
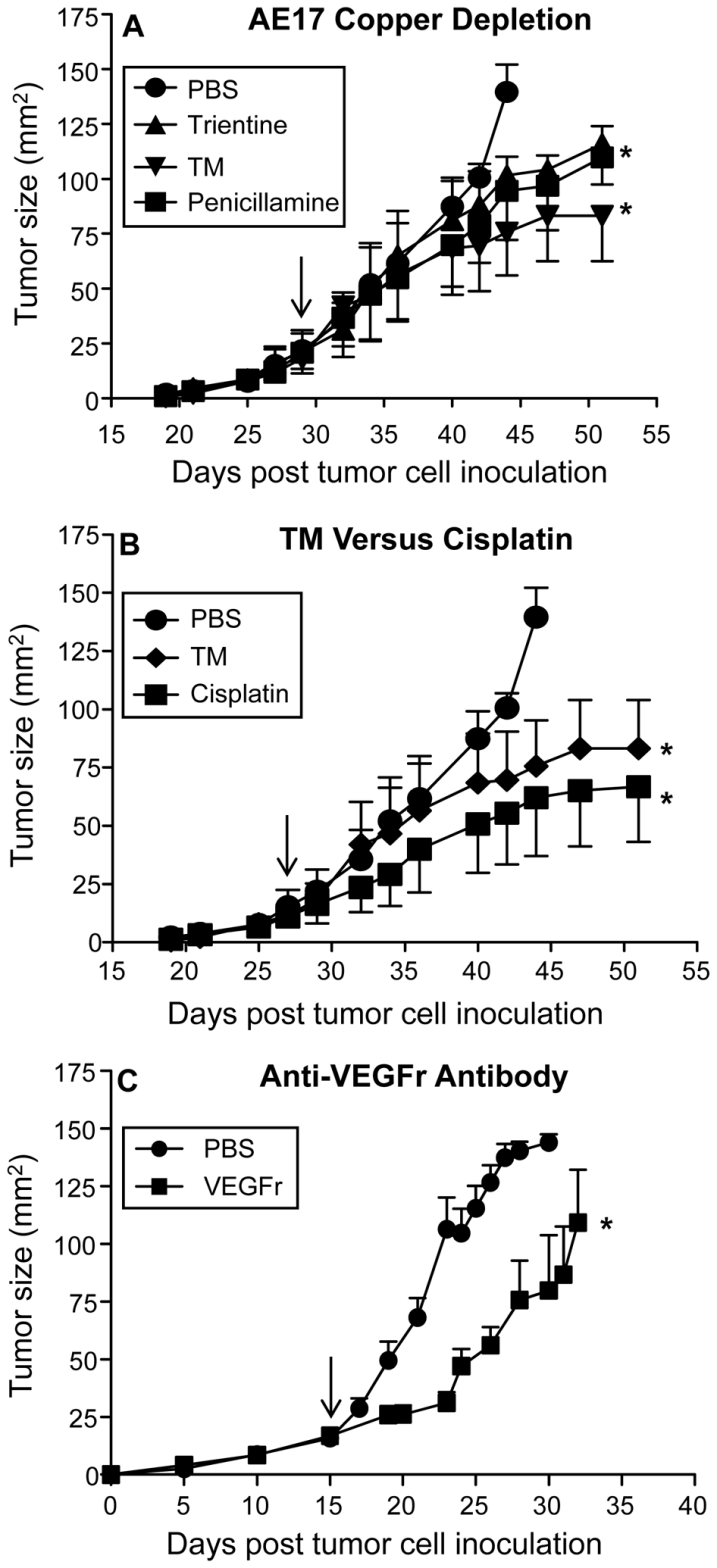
Reducing bioavailable copper slows tumor growth rate. AE17-bearing mice were given daily PBS, TM (500 µg/dose/mouse), penicillamine (2000 µg/dose/mouse) and trientine (700 µg/dose/mouse) throughout tumor growth (A). Pooled data is from one experiment (n = 4 or 5 mice/group). In a separate experiment, AE17-bearing mice were given cisplatin, TM or PBS; n = 6 mice/group (B). In another experiment AE17-bearing mice were given i.p. injections (800 µl) of PBS or 800 µg/dose/mouse anti-VEGFr antibody (C) (n = 10 mice/group). Arrow indicates when treatment was commenced. All data are shown as mean ± SEM. * p < 0.05.

We next aimed to determine if lowering bioavailable copper could be used therapeutically by commencing treatment after mesothelioma tumors have been established in vivo. To do this, the three copper depleting strategies were commenced when established tumors reached 20-25 mm^2^. All three agents slowed mesothelioma growth (Figure 3A) and tumors in trientine and TM-treated mice were significantly smaller than the controls. In a separate experiment, the in vivo anti-tumor activity of TM was compared to cisplatin, a cytotoxic agent often included in the treatment regimen for mesothelioma patients. Again, treatment with TM or cisplatin commenced when tumors reached 20-25 mm^2^. TM and cisplatin were equally efficacious to each other and each significantly slowed tumor growth (Figure 3B). Finally, in another experiment the in vivo anti-tumor activity of an anti-angiogenic strategy that prevents VEGF action by blocking the VEGR receptor (VEGFr) was examined using the rat anti-murine VEGFr monoclonal antibody (DC101). The anti-VEGFr antibody treatment, commenced when tumors reached 20-25 mm^2^, also slowed tumor growth (Figure 3C). These data show that anti-angiogenic approaches such as copper depletion and anti-VEGFr beneficially interfere with mesothelioma tumor growth.

### Prophylactically lowering bioavailable copper transiently slows mesothelioma tumor growth rate

We also aimed to determine if lowering bioavailable copper could be used prophylactically by commencing the three copper depleting strategies two weeks before mesothelioma tumors were implanted and then maintaining treatment to the end of the experiment. The copper-depleting treatments, in particular trientine, appeared to delay tumor growth for 35 days post tumor cell inoculation. After day 35 tumor growth in all copper depleting treatment groups had caught up with, or overtaken, the controls (Figure S4A).

### Saturating the tumor microenvironment with high copper levels does not accelerate tumor growth

We conducted two different approaches to see if high copper levels would promote tumor growth. Firstly, we pre-loaded copper by giving mice copper acetate in their drinking water at 500 mg/L two weeks prior to inoculation with AE17 tumor cells. Secondly, we post-loaded copper by allowing tumors to develop to 1-4 mm^2^ sized tumors before copper acetate was included in the drinking water. Tumor growth was barely perturbed in the pre-copper loading experiment and, unexpectedly, was slowed in the post-copper loading experiment.

### Copper lowering is not directly toxic to mesothelioma tumor cells or endothelial cells

**Figure 4:**
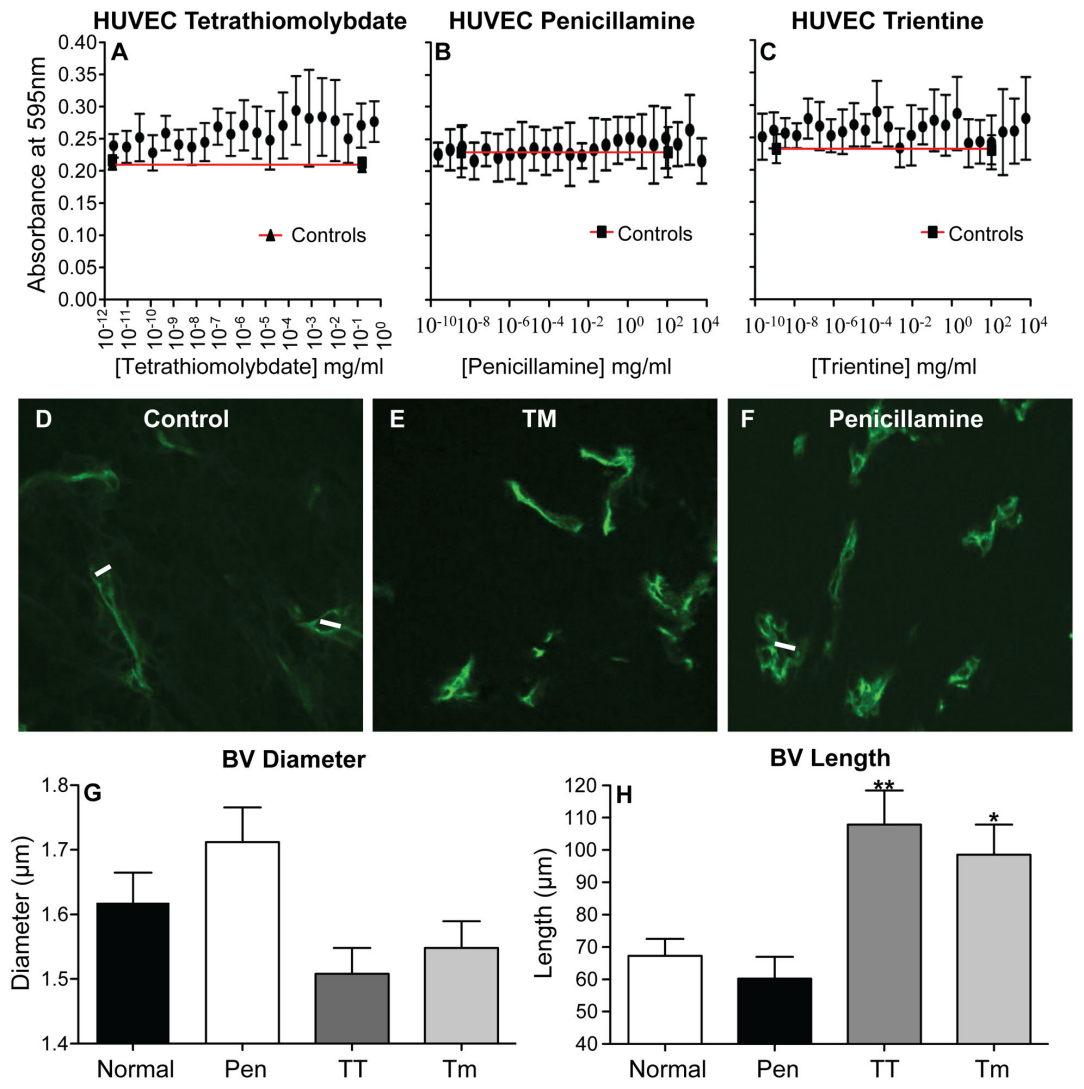
Decreasing bioavailable copper in vivo modulates tumor vessel dimensions. HUVECs (5 x 10^3^) were treated in vitro with PBS (control is shown as the black line) or log fold concentrations of tetrathiomolybdate (A), penicillamine (B) and trientine (C) and MTT assays performed 24 hrs later. Data from n = 2 experiments each with its own triplicates is shown as mean ± range. Mice treated with PBS, TM, penicillamine or trientine were sacrificed when tumors reached 100 mm^2^ and tumor-associated vessels visualized and measured on frozen tumor sections using FITC-anti-CD31 antibody and confocal imaging. Representative photographs are from individual mice that were not treated (control: D), given TM (E) or penicillamine (F). Pooled data counting a minimum of 100 cells per mouse showing tumor blood vessel (BV) diameter (G) and length (H) from control (normal mice; n = 4), penicillamine (Pen; n = 2), trientine (TT; n = 2) or Tm-treated (n = 2) mice are shown as mean ± SEM. * p < 0.05, ** p < 0.005.

MTT assays were used to determine if copper depletion affects AE17 tumor cell growth, metabolic activity and/or viability, which could account for the slowed tumor growth rates shown in Figure 3A. The results showed that TM, penicillamine and trientine did not exert a significant effect on tumor cell proliferation at the log fold concentration range examined (Figure S3A, S3B and S3C). These data suggest that copper lowering is not directly toxic to mesothelioma tumor cells, thus other mechanisms must be contributing to the slowed tumor growth. It has been suggested that copper lowering targets tumor blood vessels [6,37–39]. Therefore, HUVECs were used to determine the effect of copper lowering on normal healthy endothelial cells. HUVECs continued to proliferate in the presence of TM and trientine (Figure 4A and C) at similar levels to the controls. Treatment with penicillamine slightly increased their proliferation relative to the controls (Figure 4B).

### Copper lowering in vivo modulates tumor endothelial cells

The data above suggests that the copper chelating reagents are not toxic to endothelial cells and the penicillamine results implied the potential for endothelial cell activation. Therefore, the next series of experiments assessed the effect of copper lowering on mesothelioma-associated tumor blood vessels in vivo. Blood vessels connecting draining inguinal lymph nodes and tumors appeared to shrink in diameter in TM and trientine (TT) treated mice (not shown), similarly, the diameter of tumor-associated vessels decreased (Figure 4D, E and G), while vessel length increased (Figure 4D, E and H) in the same mice. Penicillamine appeared to increase tumor vessel diameter but not length (Figure 4F, G and H). These data suggest that blood vessel structure is being affected by copper lowering strategies.

**Figure 5:**
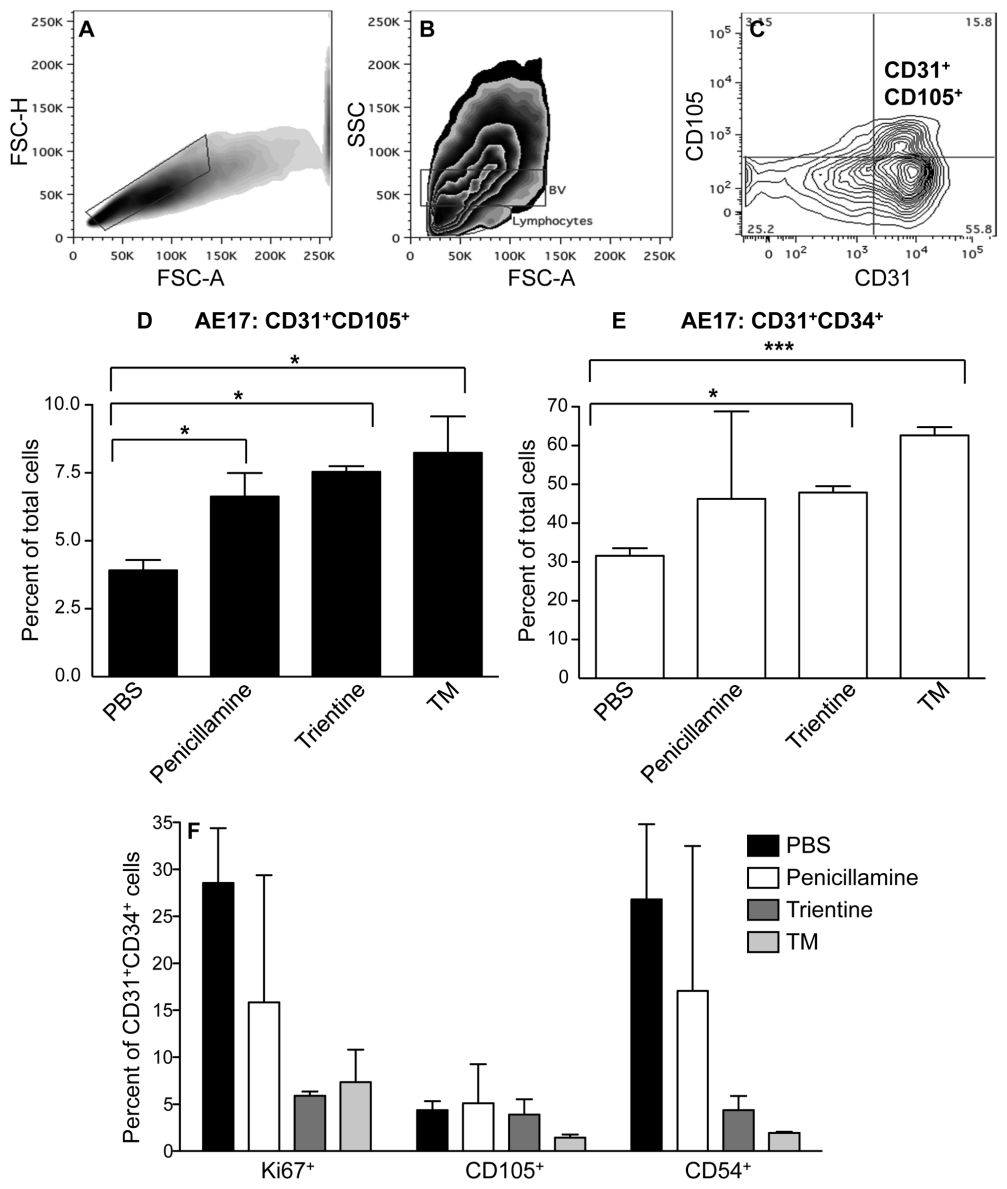
Copper lowering in vivo reduces tumor endothelia proliferation and ICAM (CD54) expression. Mice treated with PBS, TM, penicillamine or trientine were sacrificed when tumors reached 100 mm^2^ and tumor endothelia analyzed by flow cytometry. Single cells were identified by excluding doublets by gating on FSC-A versus FSC-H plots; A. Isotype controls and single stains were used to set up negative and positive regions (not shown). Lymphocytes were excluded by gating. Backgating on CD31^+^CD105^+^ revealed the FSC/SSC region associated with tumor blood vessels (BV:B). CD31^+^CD105^+^ angiogenic (C and D) or CD31^+^CD34^+^ normal (E) endothelial cells were identified, gated and quantified. The proportions of Ki67^+^, CD105^+^ and CD54^+^ cells within each gate were determined (F). Pooled data counting > 20,000 CD31^+^CD105^+^ or CD31^+^CD34^+^ cells per mouse from 2 mice/treatment group and 4 control mice is shown as mean ± SEM. * p < 0.05; *** p < 0.001.

We then hypothesized that copper lowering may normalize tumor vessels as previously described [14]. This could be identified by increasing numbers of normal endothelial cells, likely recruited from circulating precursors, and reduced endothelial cell activation, the latter driven by tumor cell-derived factors. Thus, to further examine the effect of copper lowering on tumor vessels excised tumors were disaggregated into single cell suspensions and flow cytometry used to identify angiogenic and normal endothelia [28]. Unexpectedly, all agents led to a significant increase in the proportion of total gated CD31^+^ endothelial cells that represented CD31^+^CD105^+^ angiogenic endothelial cells (Figure 5A to D) however, the overall percentages were low. TT and TM induced a significant increase in the proportions of normal CD31^+^CD34^+^ endothelial cells (Figure 5E) with a reduced activation status revealed by lower proliferation (Ki67) and lower surface ICAM/CD54 expression (Figure 5F). In contrast, normal endothelial cells continued to proliferate when exposed to penicillamine in agreement with the data shown in Figure 4B. A small number of CD31^+^CD34^+^ endothelial cells co-expressed CD105 which may indicate transition into angiogenic endothelia; the proportions of these triple positive cells were also reduced in response to copper depletion strategies (Figure 5F). The most effective agents in promoting normal CD31^+^CD34^+^ endothelial cells were TM and TT. These data imply that reducing bioavailable copper normalizes tumor vessels.

### Copper lowering promotes CD4^+^ T cell infiltration into the tumor microenvironment

**Figure 6:**
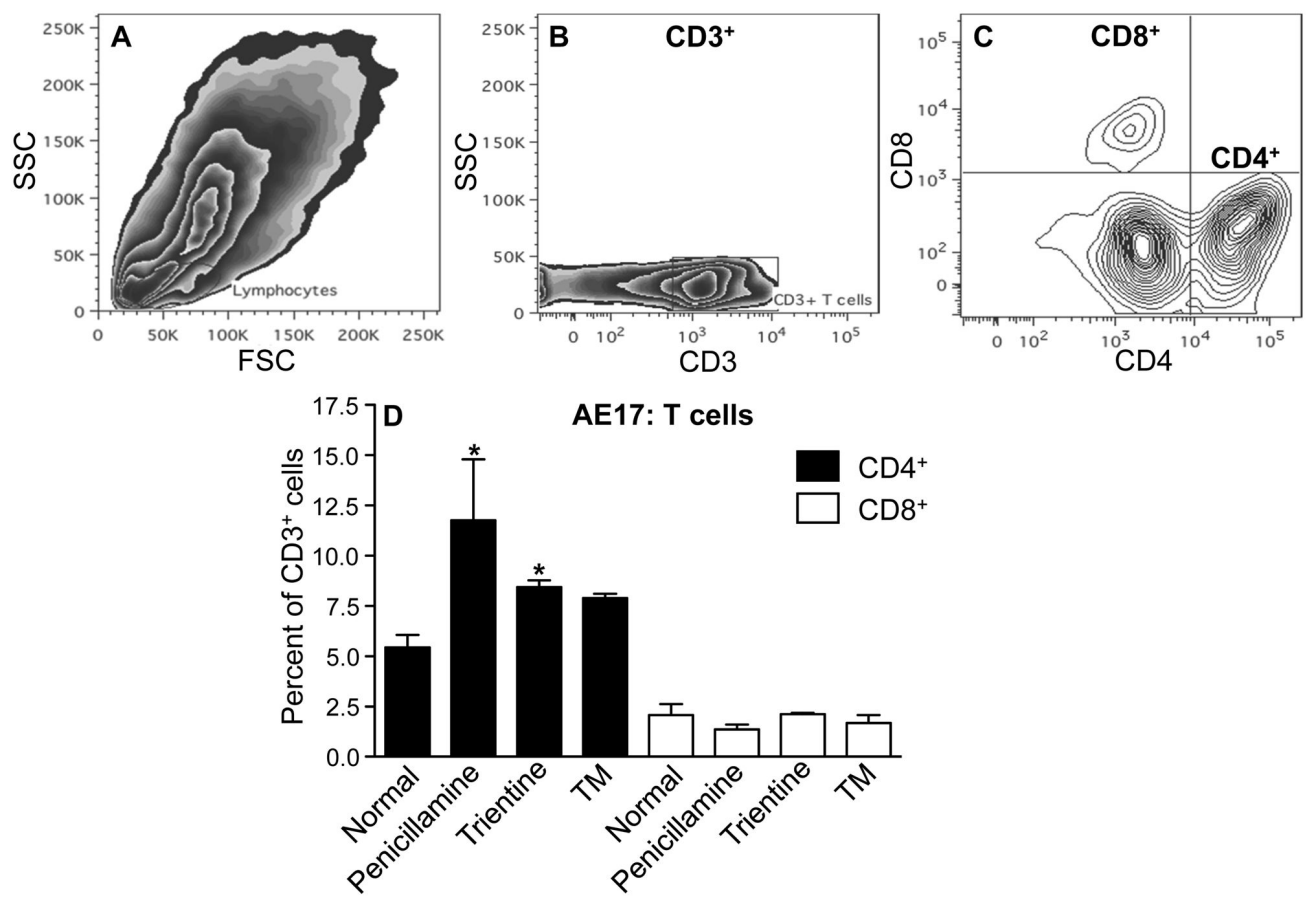
Copper lowering in vivo promotes CD4^+^ T cell infiltration. Mice treated with PBS, TM, penicillamine or trientine were sacrificed when tumors reached 100 mm^2^ and immune cell infiltration analyzed by flow cytometry. The SSC/FSC-A plot revealed the lymphocyte population (A). CD3^+^ regions were determined using the CD3-APC-Cy7 single stain (B). Triple staining identified CD3^+^CD4^+^ or CD3^+^CD8^+^ cells; representative contour plot (C). Pooled data from 2 mice/group shown as mean ± SEM (D). * p < 0.05.

Others have shown that normalized tumor vessels become permissive to immune cell infiltration [15]. Thus, we used flow cytometry to measure changes to tumor-infiltrating CD4^+^ and CD8^+^ T cells. A significant increase in CD4^+^ T cells was seen in AE17 tumors treated with penicillamine and trientine (Figure 6A to D). An apparent but not statistically significant increase in CD4^+^ cells was also observed in TM treatment. No change was observed in CD8^+^ T cell levels.

## Discussion

To our knowledge, no previous studies have examined the role of copper in mesothelioma tumors. This study found that murine mesothelioma tumors rapidly accumulate copper as they progress. The biggest change was seen in small early stage tumors. It is interesting to note that copper concentrations in organs, such as the liver, show a similar pattern of high concentrations during fetal growth; these levels then decline due to their gradual use and dispersal throughout the growing tissue [24,40]. Copper’s role in angiogenesis, and therefore in organ development, may explain the rapid copper acquisition seen in fetal tissues. Thus, copper may play an important role in the early stages of mesothelioma tumor development by supporting angiogenesis, as described in other tumors [41].

Reduction of bioavailable copper was tested using copper lowering agents combined with copper-depleted drinking water. We used copper-depleted water because it was relatively easy to remove copper from water and it ensured that changes in drinking rates would not affect our results. However, the food contained copper and the average mouse consumes about 4.5 g of food per day [42] which equates to 72µg copper. Nonetheless, despite copper replenishment by food penicillamine and trientine reduced liver copper levels at all doses examined. In contrast, TM showed a transient increase in liver copper levels before a rapid decrease, which may reflect copper being mobilized from other organs to the liver before excretion via the bile. Copper bound to ceruloplasmin increases angiogenic activity and correlates with tumor progression [43]. Thus, in clinical trials the first goal of anti-cancer copper reduction is to reduce ceruloplasmin levels to 20% of the patient’s normal baseline [27]. Penicillamine and TM were more effective at lowering ceruloplasmin levels in the blood than trientine. Optimal doses were chosen based on the lowest dose that lowered liver copper and ceruloplasmin whilst maintaining a threshold level of copper required for essential body functions. The latter was indicated by monitoring the general health status of mice. Note that future studies will use a copper depleted diet to further evaluate our work.

Limiting bioavailable copper has been shown to slow tumor growth in other cancers [4–12]. In this study copper lowering with TM and trientine significantly slowed murine AE17 mesothelioma tumor growth rate. Nonetheless, TM and trientine treatment did not offer long-term survival with all mice eventually succumbing to tumors. These data were similar to the standard platinum chemotherapy, cisplatin, as well as to an anti-angiogeneic strategy that blocks the function of VEGF, i.e. both as monotherapies induced transient tumor regression before tumor progression. It is possible that combining decreasing bioavailable copper with chemotherapy and/or other vascular targeting strategies may provide an improved outcome. It should be noted that the anti-angiogenic effect of copper deficiency, induced by TM at least, is via suppression of NF _k_B including NF _k_B-mediated transcription of pro-angiogenic factors [5]. Therefore, some chemotherapeutic agents, such as doxorubicin, but not all, can be combined with copper depletion. Indeed, we now have preliminary data showing that combining copper depletion treatment with cisplatin is antagonistic (data not shown). The only clinical trial in mesothelioma patients reported to-date was a combination therapy in which TM therapy was given post surgery, and the data showed that the time to disease progression was slowed by 10 months [11]. As the current survival time for patients with mesothelioma is 12 months post diagnosis any therapy that increases survival time could make a considerable difference in the lives of these patients. Further studies are required to address combination with other treatment modalities.

Our studies showed that commencing copper depletion two weeks prior to tumor cell inoculation transiently delayed mesothelioma tumor growth for 31 to 35 days depending on the treatment used. Similarly, depleting copper after mesothelioma tumors were established but at a time when the tumors appeared to be dispersing rather than rapidly acquiring copper led to delayed tumor growth. Previous work has shown that tumors cannot grow past the angiogenic switch in a copper depleted environment [5]. However, some trials have shown that copper depletion has little affect on tumor growth [44,45], and it is possible that once tumors are fully angiogenic depleting copper will have little effect. One possible interpretation of our data is that the mesothelioma tumors did not reach true angiogenesis and until around day 31 and therefore remained susceptible to copper depletion until that time. These data contrast to our data generated in concurrent and identical experiments we conducted using the Lewis Lung carcinoma cell line. In this lung cancer model, copper depletion prior to tumor cell inoculation was more effective in delaying tumor growth (data not shown) than in the mesothelioma model. Yet and in contrast to the mesothelioma model, copper depletion after LL tumors were established was not effective (data not shown), implying a rapid angiogenic switch in the LL model. We did not measure copper accumulation in mesothelioma or LL tumors developing in an environment initially depleted of copper, nor did we release tumors from copper depletion. This will be the subject of future studies.

We also attempted to copper load mice to accelerate in vivo tumor growth in both models. Pre-loading with copper had little impact on mesothelioma tumor growth whilst copper loading post tumor cell inoculation appeared to be toxic to the mesothelioma microenvironment as copper-loaded tumors grew at a slower rate. Again, this contrasted to the LL model in which copper loading promoted tumor growth (data not shown). The data are difficult to interpret, however one possibility is that copper levels were already at the highest possible physiological concentrations required for mesothelioma, but not LL, angiogenesis and that increased copper levels become toxic to mesothelioma development. These data suggest that different models may have different copper requirements, as the mouse models are both in C57BL/6 mice and were s.c. implanted using the same number of tumor cells.

Copper depletion induced structural and functional changes in tumor-associated blood vessels. Changes to the length and diameter of tumor blood vessels coincided with a significant increase in the proportion of endothelial cells that were less activated, i.e. reduced ICAM (CD54) and lower proliferative levels. These data suggest that vessel normalization had been induced. It is possible that these normalized cells represent newly recruited (CD31^+^CD34^+^) endothelial cells that were progressing toward an angiogenic phenotype; the acquisition of CD105^+^ supports this hypothesis. These data are in agreement with an early study using a rabbit brain tumor model in which copper lowering reduced tumor vessel width, as well as the proliferative rate of tumor endothelia, to similar levels seen in healthy brain vessels [12]. Similarly, a recent study has shown that copper plays an important role in vascular inflammation, and that copper chelation with TM may have value as an anti-inflammatory or anti-atherogenic agent [26]. In those studies mice treated with TM demonstrated significant inhibition of lipopolysaccharide-induced inflammatory gene transcription and protein levels of VCAM-1, ICAM-1 and monocyte chemotactic protein-1 in the aorta and heart. Thus, reducing bioavailable copper may return activated/inflamed angiogenic endothelial cells to a more normal state [13].

A significantly higher CD4^+^, but not CD8^+^, T cell infiltrate was seen in mesothelioma tumors undergoing copper lowering therapy. These data imply that reducing copper levels renders blood vessels to become permissive to CD4^+^ T cell infiltration. The reasons for this selective recruitment are unclear. One possibility is changes to expression of specific adhesion molecules. An interesting molecule that may control CD40L^+^CD4^+^ T cell adhesion to endothelium is CD40 which can be expressed at higher levels by tumor endothelial cells relative to endothelia in normal tissues [46,47]. The effect of copper lowering on CD40 expression by tumor vessels is as yet unknown and further studies are required. It is also possible that the CD4^+^ T cells recruited into tumors represent a T cell subset such as the CD4^+^CD7^-^ subset which can preferentially adhere to endothelial cells via ICAM and E-selectin (CD62E), the ligand of which, cutaneous lymphocyte-related antigen (CLA), is highly expressed in CD4^+^CD7^-^ T cells [48]. Further studies are required to elucidate the molecular mechanisms underlying CD4^+^ T cell selection and CD4^+^ T cell function in the tumor microenvironment.

Here we show that copper is rapidly sequestered into developing mesothelioma tumors and that therapeutic copper removal from organs, the circulation and the tumor itself slows tumor growth. Our findings suggest copper lowering modulates tumor blood vessels such that they are transiently ‘normalized’ to promote immune cell infiltration into the tumor microenvironment. These data suggest combination with immunotherapy should be investigated. We propose that copper lowering is a potentially useful and relatively non-toxic anti-mesothelioma strategy that slows tumor growth to provide a window of opportunity for inclusion of other treatment modalities to improve outcomes for mesothelioma patients.

## Supporting Information

Figure S1 **Tap water is a significant source of copper.**
Tap water from the animal facility was analyzed for Cu, before and after aqua pure (filtered) or Milli-Q filtration (A); one sample, data shown as mean of each triplicate. Mice from the same facility were given either tap or Milli-Q water ad libitum for 10 days, before their organs were analyzed for Cu levels; pooled data from 6 mice/group is shown as mean ± SEM (B).
(TIF)

Figure S2 **Mesothelioma tumors do not affect copper levels in other organs.**
AE17 tumor-bearing mice were sacrificed at varying timepoints and liver (A), kidney (B), lung (C) and spleen (D) Cu levels measured. Pooled data from the same four experiments described for Figure 2 is shown as mean ± SEM.
(TIF)

Figure S3 **Copper lowering is not toxic to mesothelioma cells in vitro.**
AE17 tumor cells (5 x 10^3^) were treated in vitro with log fold concentrations of tetrathiomolybdate (A), penicillamine (B) and trientine (C) and MTT assays performed 24 hrs later. Data was normalised to control cells (black line). Pooled data from 3 experiments each with its own triplicates is shown as mean ± SEM.
(TIF)

Figure S4 **Copper lowering prior to tumor cell inoculation slows tumor growth.**
Mice received daily i.p. injections of penicillamine (2000µg), trientine (700µg) or TM (200µg) for 2 weeks (starting day -14; pre Cu depletion: A) prior to AE17 tumor cell inoculation (on day 0), copper lowering continued until the mice were culled (n = 6 mice per experimental group). Copper loading was achieved by giving mice copper acetate in their drinking water at 500 mg/L two weeks prior to inoculation with AE17 tumor cells (n = 6 mice/group; pre-loading: B), or by allowing tumors to develop to 1-4 mm^2^ sized tumors before copper acetate was continuously included in the drinking water (n = 5 mice/group; post-loading: C). Data shown as mean ± SEM.
(TIF)
