# Supplementary figures and images for: Rapid Copper Acquisition by Developing Murine Mesothelioma: Decreasing Bioavailable Copper Slows Tumor Growth, Normalizes Vessels and Promotes T Cell Infiltration
Source: PLoS One. 2013 Aug 27;8(8):e73684. doi: 10.1371/journal.pone.0073684 (PMC3754934; doi:10.1371/journal.pone.0073684)

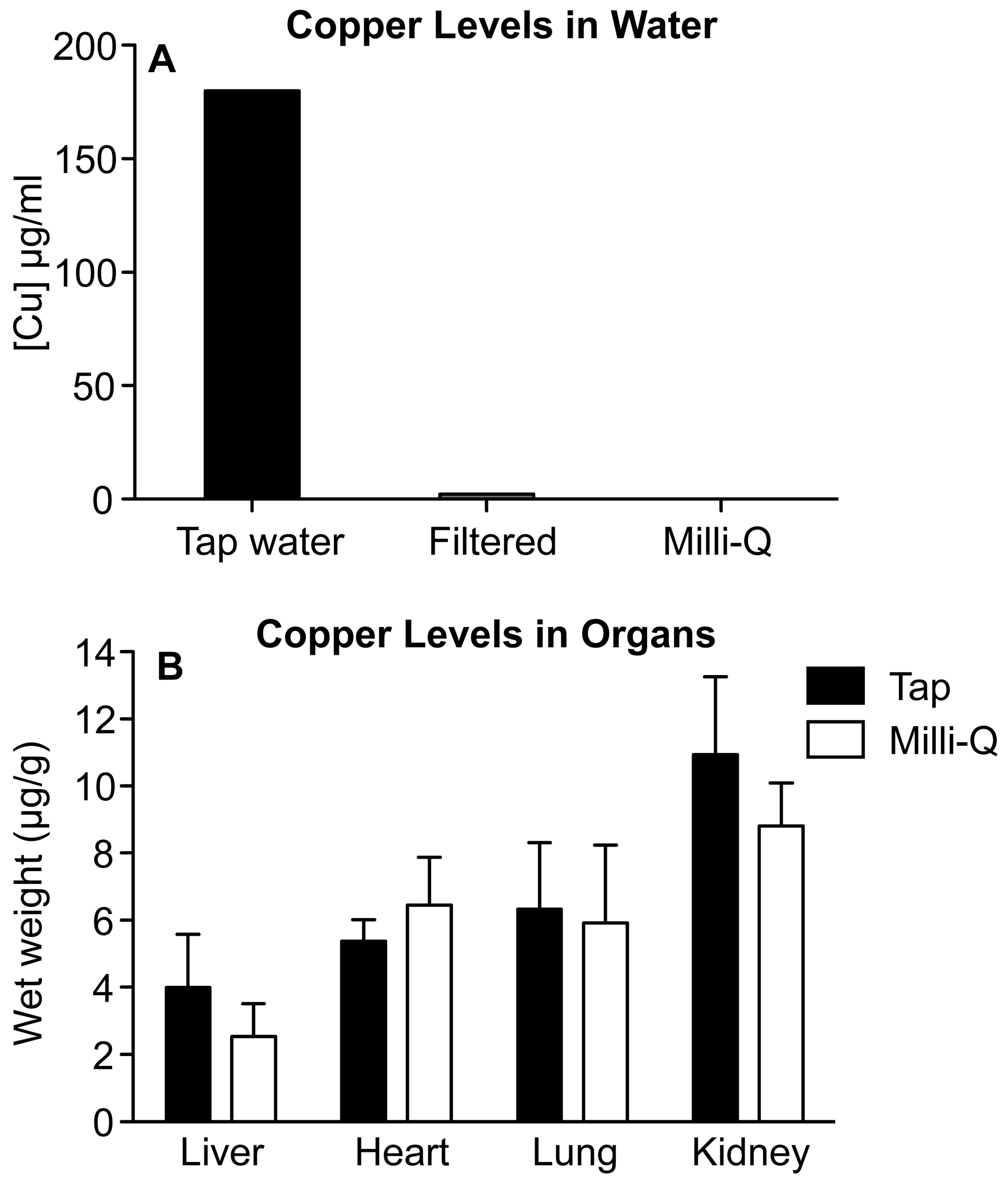

Supplement: Figure S1 — Tap water is a significant source of copper. Tap water from the animal facility was analyzed for Cu, before and after aqua pure (filtered) or Milli-Q filtration (A); one sample, data shown as mean of each triplicate. Mice from the same facility were given either tap or Milli-Q water ad libitum for 10 days, before their organs were analyzed for Cu levels; pooled data from 6 mice/group is shown as mean ± SEM (B). (TIF) [file pone.0073684.s001.tif]
